# The Research Progress of Exosomes in Osteoarthritis, With Particular Emphasis on the Mediating Roles of miRNAs and lncRNAs

**DOI:** 10.3389/fphar.2021.685623

**Published:** 2021-05-21

**Authors:** Chenggui Miao, Wanwan Zhou, Xiao Wang, Jihong Fang

**Affiliations:** ^1^ Department of Pharmacology, School of Integrated Chinese and Western Medicine, Anhui University of Chinese Medicine, Hefei, China; ^2^ Department of Pharmacy, School of Life and Health Sciences, Anhui University of Science and Technology, Fengyang, China; ^3^ Institute of Prevention and Treatment of Rheumatoid Arthritis of Chinese Medicine, Anhui University of Chinese Medicine, Hefei, China; ^4^ Department of Clinical Nursing, School of Nursing, Anhui University of Chinese Medicine, Hefei, China; ^5^ Department of Nursing, Anhui Provincial Children’s Hospital, Affiliated to Anhui Medical University, Hefei, China; ^6^ Department of Orthopedics, Anhui Provincial Children’s Hospital, Affiliated to Anhui Medical University, Hefei, China

**Keywords:** exosomes, non-coding RNA, long noncoding RNA, osteoarthritis, microRNA

## Abstract

Osteoarthritis (OA) is a kind of degenerative disease, which is caused by many factors such as aging, obesity, strain, trauma, congenital joint abnormalities, joint deformities. Exosomes are mainly derived from the invagination of intracellular lysosomes, which are released into the extracellular matrix after fusion of the outer membrane of multi vesicles with the cell membrane. Exosomes mediate intercellular communication and regulate the biological activity of receptor cells by carrying non-coding RNA, long noncoding RNAs (lncRNAs), microRNAs (miRNAs), proteins and lipids. Evidences show that exosomes are involved in the pathogenesis of OA. In view of the important roles of exosomes in OA, this paper systematically reviewed the roles of exosomes in the pathogenesis of OA, including the roles of exosomes in OA diagnosis, the regulatory mechanisms of exosomes in the pathogenesis, and the intervention roles of exosomes in the treatment of OA. Reviewing the roles of exosomes in OA will help to clarify the pathogenesis of OA and explore new diagnostic biomarkers and therapeutic targets.

## Introduction

Exosomes are small membrane bubbles (40–150 nm) containing complex RNAs and proteins. Many cells can secrete exosomes in physiological and pathological states. They are mainly derived from the vesicles formed by the collapse of lysosomal particles, which are released into extracellular matrix after fusion of the outer membrane and cell membrane (Zhang and Yu, 2019; Kalluri and LeBleu, 2020).

Almost all types of cells can secrete exosomes, which naturally exist in body fluids, including blood, saliva, urine, cerebrospinal fluid and milk. The precise molecular mechanisms of their secretion and uptake, composition, “carrier” and corresponding functions has just begun to be studied (Meldolesi, 2018). Exosomes are considered as specific vesicles and participate in intercellular communication. There is a growing interest in the study of exosomes, whether to study their functions or to understand how to use them in the development of minimally invasive diagnosis (Mashouri et al., 2019).

When exosomes are secreted from host cells into receptor cells, exosomes can regulate the biological activity of receptor cells by carrying proteins, nucleic acids and lipids (Gonda et al., 2019). Exosome mediated intercellular communication mainly through the following ways. First, exosome membrane proteins can bind to target cell membrane proteins and activate signal pathways in target cells. Second, in the extracellular matrix, exosome membrane proteins can be cleaved by proteases. The cleaved fragments can act as ligands to bind to receptors on the cell membrane, thus activating intracellular signaling pathways. Thirdly, the exosome membrane can fuse directly with the target cell membrane, and non-selectively release proteins, mRNAs, microRNAs (miRNAs) and long noncoding RNAs (lncRNAs) (Familtseva et al., 2019; Jiang et al., 2019).

**FIGURE 1:**
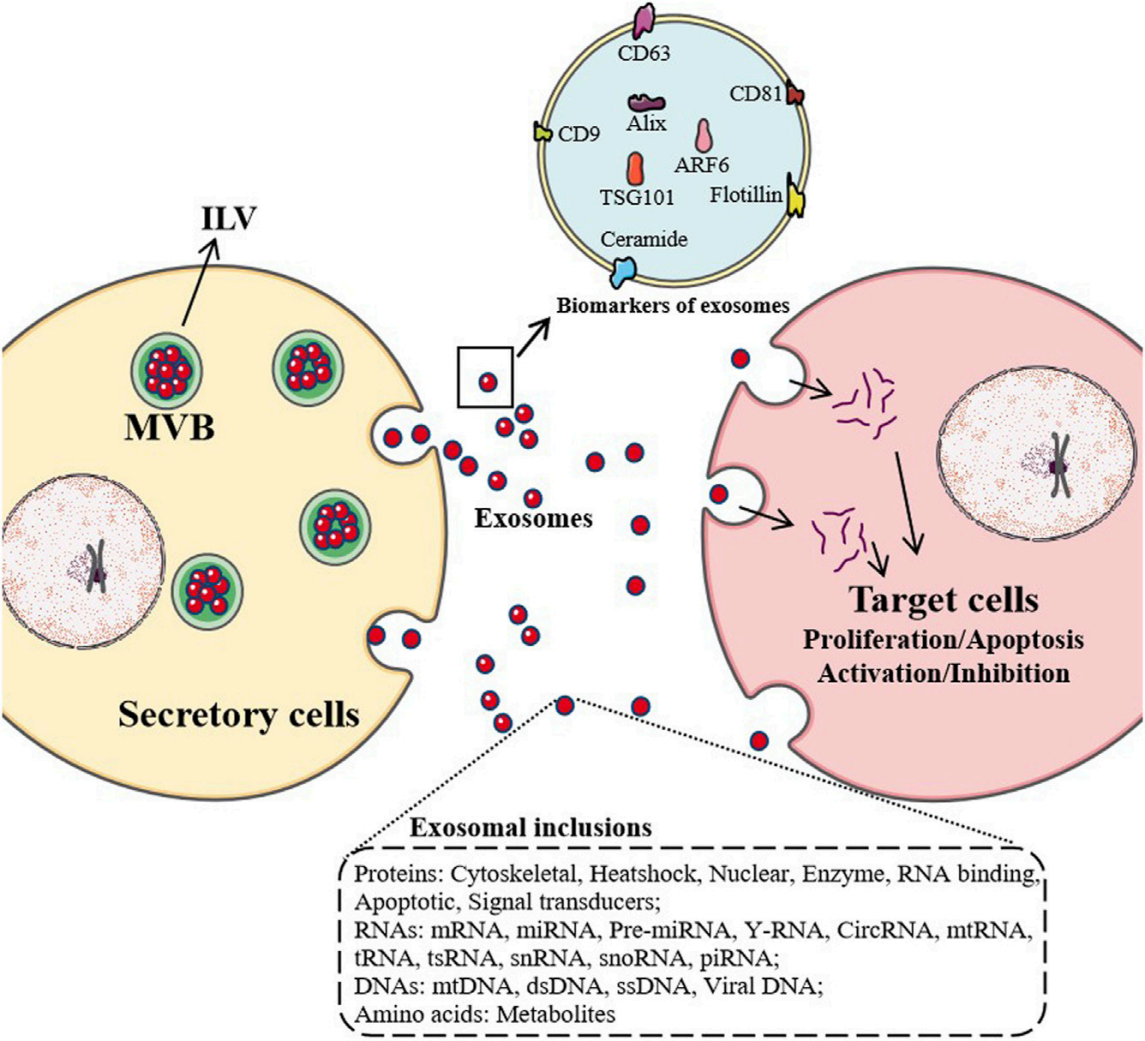
Maturation and secretion mechanisms of exosomes. After most endosomes mature to multivesicular bodies (MVB) or late endosomes, their contents, RNAs, proteins, lipids are packaged as intraluminal vesicles (ILV) in MVB. With the fusion of MVB and cell membrane, ILV are released as exosomes and enter target cells by endocytosis, which affect the physiological and pathological mechanisms of target cells, such as proliferation, apoptosis, activation and inhibition.

When exosomes are first discovered, they are considered as a way for cells to excrete waste. Nowadays, with a large number of studies on their biological sources, material composition, transportation, intercellular signal transduction, and distribution in body fluids, exosomes have been found to have a variety of functions (Shan et al., 2019). Exosomes can participate in immune response, antigen presentation, cell migration, cell differentiation, tumor invasion. Studies have shown that exosomes participate in the pathological mechanisms of osteoarthritis (OA), and promote the pathological development of OA (Chen B. Y. et al., 2019; Zhou Q. F. et al., 2020) (Figure 1).

OA is a kind of degenerative disease, which is caused by many factors such as aging, obesity, strain, trauma, congenital joint abnormalities, joint deformities (Hunter and Bierma-Zeinstra, 2019; Kan et al., 2019). The disease is more common in the middle-aged and elderly people, and occurs in the weight-bearing joints and joints with more activity (such as cervical spine, lumbar spine, knee joint, hip joint, etc.). Excessive weight bearing or the use of these joints can promote the occurrence of degenerative changes. The clinical manifestations includes joint swelling, joint stiffness, joint movement limitation (Sacitharan, 2019; Abramoff and Caldera, 2020).

The main symptom is joint pain, often rest pain, which is manifested as pain after rest. After a moment of activity, the pain is relieved, but after too much activity, the pain is aggravated (Mandl, 2019). Another symptom is joint stiffness, which often occurs in the morning or after the joint maintains a certain position for a long time in the daytime. Joint swelling can be seen in the affected joints. There is a sense of friction or “click” sound during activity. Muscle atrophy and joint deformity can be found in severe cases (O'Neill and Felson, 2018; Geyer and Schönfeld, 2018).

At present, mesenchymal stem cells (MSCs) are considered to be related to the development of OA. Some people believe that the paracrine of nutritional factors, including exosomes mediated secretion, plays an important role in MSC based OA treatment mechanisms (Colombini et al., 2019; Song et al., 2020). There is evidence that the paracrine of exosomes may play important roles in the repair of joint tissue. Exosomes isolated from various stem cells contribute to tissue regeneration of heart, limbs, skin and other tissues, and exosomes derived from MSCs may inhibit the pathological development of OA (Mianehsaz et al., 2019).

In this work, we reviewed the role of exosomes in the pathogenesis of OA, which will help to clarify the pathogenesis of OA and explore new diagnostic biomarkers and therapeutic targets.

## Circulating Exosomes as Diagnostic Biomarkers of Osteoarthritis

### Exosomes in Peripheral Blood

Exosomes may play important roles in OA diagnosis, and the miRNAs, lncRNAs and other inclusions in exosomes may be new biomarkers for OA diagnosis (Otahal et al., 2020). In OA, miR-193b-3p regulates the chondrogenesis and chondrocyte metabolism by targeting the histone deacetylase 3 (HDAC3). The expression of miR-193b-3p increases in chondrogenic and hypertrophic human MSCs, but decreases in degenerative cartilage. The level of plasma exosomal miR-193b-3p in OA patients is significantly lower than that in control group, suggesting that the exosomal miR-193b-3p may be a new diagnostic marker for OA (Meng et al., 2018).

Plasma exosomes of OA patients can induce OA chondrocytes to express cartilage genes and inhibit the release of inflammatory cytokines. This highlights the potential of plasma exosome inclusions as regulators of extracellular matrix metabolism and inflammation, and may be candidates for a new approach of cell-free therapy and diagnosis of OA (Chang et al., 2018).

Exosomes in plasma of OA patients have been found to have new potential in relieving knee osteoarthritis. The mechanisms may be that exosome inclusions promote the chondrocyte proliferation and inhibit the chondrocyte apoptosis through the Wnt/β-catenin signaling pathway. It also suggests the feasibility of exosome inclusions as diagnostic markers for OA (Yuan et al., 2016). In OA chondrocytes treated by IL-1β, platelet-rich plasma (PRP) exosomes can inhibit the release of TNF-α, promote the proliferation of OA chondrocytes, and significantly reduce the apoptosis rate of OA chondrocytes. PRP exosomes, as carriers containing PRP derived growth factors, provide a new way for the diagnosis of OA (He et al., 2020).

The levels of lncRNA PVT1 and high mobility groupprotein B1 (HMGB1) are up-regulated, while the level of miR-93-5p is down-regulated in serum and LPS induced C28/I2 cells. PVT1 deletion can reverse the decrease of cell viability, increase of apoptosis and inflammation induced by LPS in C28/I2 cells. PVT1 regulates the expression of HMGB1 through miR-93-5p. Inhibition of miR-93-5p can eliminate the apoptosis, inflammatory response and collagen degradation of C28/I2 cells mediated by PVT1 silencing. The increase of HMGB1 reverses the up-regulation of miR-93-5p mediated apoptosis and inflammation of C28/I2 cells. In addition, PVT1 regulates the TLR4/NF-κB pathway through miR-93-5p/HMGB1 axis. Obviously, PVT1 gene knockout by exosomes can inhibit the pathological development of OA through miR-93-5p mediated HMGB1/TLR4/NF-κB pathway, and these exosomal inclusions may be new diagnostic markers for OA (Xue et al., 2019; Meng et al., 2020; Sun et al., 2020).

### Exosomes in Synovial Fluid

There are gender differences in exosome proteins in synovial fluid of patients with OA (Kim et al., 2020). Studies have shown that exosome-derived miRNAs in synovial fluid of OA patients were changed, and these changes were gender specific. For example, female OA specific miRNAs targeted the estrogen responsive toll-like receptor (TLR) signaling pathway. After OA derived exosomes treatment, the expression of anabolic genes decreased, catabolic genes were up-regulated, and the expression of inflammatory genes also increased significantly (Kolhe et al., 2017).

In the synovial fluid exosomes of female OA, the levels of haptoglobin, orosomucoid and ceruloplasmin are up-regulated, while the level of apolipoprotein is down-regulated. In the synovial fluid exosomes of male OA, the levels of β-2-glycoprotein and complement component five protein are significantly up-regulated, while the level of Spt-Ada-Gcn5 acetyltransferase (SAGA)-related factor 29 is down-regulated. There are gender differences in synovial fluid exosome protein content in OA patients, which indicates that these differentially expressed proteins may be new diagnostic markers for OA (Kolhe et al., 2020).

Synovial fluid contains various cytokines, and most of them are not only in free form, but also enriched in exosomes (Carlson et al., 2018). Compared with the cytokine spectrum of synovial fluid, the exosomes of patients with end-stage OA have more cytokine content, especially chemokines. Synovial fluid derived exosomes recruit inflammatory cells, inhibit cartilage proliferation and promote joint degeneration. Synovial fluid microenvironment and exosomes mediated intercellular communication provide a new perspective for OA pathological research, and these exosomal cytokines may be new diagnostic biomarkers for OA (Gao et al., 2020).

Exosomal lncRNAs in synovial fluid are valuable in the differential diagnosis of early and late-stage OA. For example, the levels of exosomal lncRNAs in early OA and late-stage OA synovial fluid are significantly higher than that in control group. The expression of lncRNA PCGEM1 in patients with late-stage OA is significantly higher than that in patients with early OA, and the expression of PCGEM1 in early OA is significantly higher than that in control group. Exosomal PCGEM1 may be a powerful indicator to differentiate early OA from late-stage OA (Zhao and Xu, 2018).

## Exosomes in Osteoarthritis Pathogenesis

**FIGURE 2:**
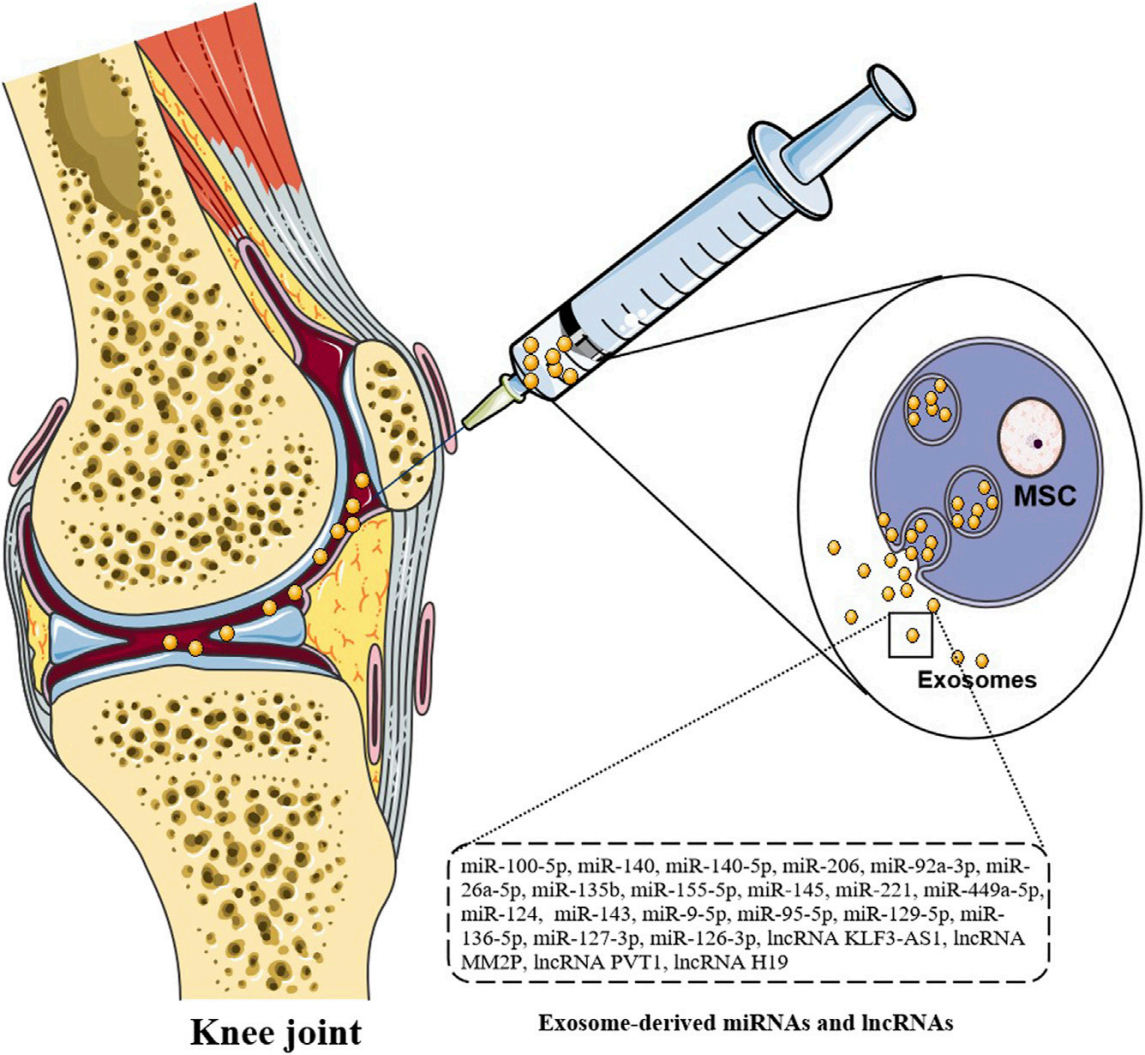
Injection therapy of exosomes. Direct injection of exosomes carrying miRNAs and lncRNAs can significantly down-regulate arthritis score, inhibit FLS proliferation and invasion, and reduce inflammatory response and joint damage.

Exosomes from various tissues play important roles in the pathogenesis of OA. IL-1β induced MSCs exosomes have obvious anti-inflammatory activity in OA sw982 cells. The roles of IL-1β in inducing MSC-derived exosomes is mediated by miR-147b, leading to the inhibition of NF-κB pathway (Kim et al., 2021). By comparing the expression of miRNAs in the exosomes of human bone marrow MSCs with that without cartilage induction, 141 differentially expressed miRNAs are found, including 35 up-regulated miRNAs, such as miR-92a, miR-193a-5p, miR-320c, miR-1246, miR-1290, 106 down -regulated miRNAs, such as miR-377-3p and miR-6891-5p. MiR-320c in the induced exosomes promotes the proliferation of OA chondrocytes and down-regulates the MMP13 expression more than that in control group. These miRNAs can induce cartilage and may play important roles in cartilage regeneration and the final treatment of OA (Sun et al., 2019) (Figure 2).

### Exosomes Derived From Synovial Mesenchymal Stem Cells

Exosomes derived from OA synovial MSCs with miR-140-5p overexpression are effective for OA treatment. Wnt5a and wnt5b in exosomes can activate the Yes-associated protein (YAP) through the Wnt signaling pathway and promote the proliferation and migration of chondrocytes. The side effect is significantly reduction of the ECM secretion, and the high expression of miR-140-5p blocks this side effect. *In vivo*, Exosomes derived from miR-140-5p-overexpressing human synovial MSCs successfully prevent the OA in model rats (Tao et al., 2017). MiR-129-5p in human synovial MSC exosomes attenuates the IL-1β induced OA by targeting the HMGB1. In OA patients and IL-1β induced chondrocytes, miR-129-5p is decreased, while HMGB1 is significantly up-regulated. MiR-129-5p targets the 3′UTR of HMGB1 and inhibits the up-regulation of HMGB1. Exosomes rich in miR-129-5p can significantly reduce the inflammatory response and apoptosis of chondrocytes, while exosomes lacking miR-129-5p increases the inflammatory response and apoptosis of chondrocytes (Qiu et al., 2021).

Synovial fluid derived MSCs transplantation is an effective method to treat OA cartilage degeneration. kartogenin (KGN) is a small molecule that can induce MSCs to differentiate into chondrocytes *in vitro* and *in vivo*. It controls the chondrogenic differentiation of transplanted MSCs. However, the poor water solubility of KGN limits its clinical application. The exosomes containing KGN can effectively solve this technical problem. MSCs binding peptide E7 is fused with exosome membrane protein Lamp 2b to produce exosomes with surface display E7 peptide (E7 exo). The E7 exo containing KGN could effectively enter MSCs, and the degree of cartilage differentiation is higher than that of KGN alone or without E7 exo (Xu et al., 2021). The E7 exo containing KGN pretreated bone marrow MSCs have higher cartilage repair efficiency, stronger cartilage matrix formation and less degradation than those from bone marrow MSCs (Liu et al., 2020).

In exosomes derived from synovial fibroblasts, overexpressed miR-126-3p inhibits the chondrocyte inflammation and cartilage degradation in OA model rats, which may have certain therapeutic value for OA patients (Zhou et al., 2021). MiR-26a-5p is low expressed in OA patients and synovial fibroblasts treated with IL-1β, while PTGS2 is high expressed. PTGS2 is a direct target of miR-26a-5p. Overexpression of miR-26a-5p alleviates the injury of synovial fibroblasts by inhibiting the PTGS2. Obviously, overexpression of miR-26a-5p inhibits the injury of synovial fibroblasts in OA through the PTGS2, which is of great significance for the treatment of OA (Jin et al., 2020b).

Exosomal lncRNA H19 in synovial fibroblasts attenuates the progression of OA through miR-106b-5p/TIMP2 axis. Cartilage repair mediated by exosomes is characterized by increased cell viability and migration as well as reduced matrix degradation. In the process of exosomes mediated cartilage repair, the enhancement of cell proliferation and migration is related to the regulation of miR-106b-5p/TIMP2 axis. Transfection of miR-106-5p mimics in chondrocytes significantly reduces the cell proliferation and migration, promotes the matrix degradation, increases the expression of MMP13 and ADAMT5, and decreases the expression of COL2A1 and ACAN in chondrocytes. TIMP2 is directly regulated by miR-106-5p. This suggests that H19 may promotes the chondrocyte proliferation and migration and inhibits the degradation of OA matrix by targeting miR-106b-5p/TIMP2 axis in OA pathogenesis (Tan et al., 2020).

### Exosomes Derived From Bone Marrow Mesenchymal Stem Cells

Exosomal lncRNA KLF3-AS1 from human bone marrow MSCs can be used as an effective therapeutic molecule for OA patients. KLF3-AS1 is significantly enriched in the exosomes of MSCs. The exosomal KLF3-AS1 inhibits the apoptosis of chondrocytes and improves the cartilage injury induced by IL-1β. KLF3-AS1 may be a potential therapeutic target for OA (Liu et al., 2018b).

In OA model mice induced by collagenase, bone marrow MSC-derived exosomes increase the chondrogenic genes type II collagen alpha 1 (Col2a1) and aggrecan, decrease the markers of chondrocyte hypertrophy MMP-13 and runt-related transcription factor 2 (Runx2), and attenuate the IL-1β induced inhibition of chondrocyte proliferation. Exosomes derived from KLF3-AS1-overexpressing-MSCs ameliorate the IL-1β induced chondrocyte injury. Interestingly, KLF3-AS1 promotes the GIT1 expression by adsorbing the miR-206. Thus bone marrow MSC-derived exosomes promote the osteoarthritis chondrocyte proliferation and inhibit the apoptosis through the KLF3-AS1/miR-206/GIT1 axis (Liu et al., 2018a).

MiR-127-3p is enriched in bone marrow MSC-derived exosomes, and bone marrow MSC-derived exosomes inhibit the IL-1β induced chondrocyte injury. MiR-127-3p can inhibit the CDH11 of chondrocytes, thus blocking the activation of Wnt/β-catenin pathway and alleviating the damage of OA chondrocytes (Dong et al., 2021).

Mao et al. (2018b) investigated the expression of miR-92a-3p in human bone marrow MSC chondrogenesis model and OA primary human chondrocytes (PHCs) *in vitro*. Interestingly, the expression of miR-92a-3p increased in MSC chondrocytes and decreased in OA chondrocytes. MSC-miR-92a-3p-exos promoted the cartilage proliferation and matrix gene expression of MSCs and PHCs, while MSC-anti-miR-92a-3p-exos inhibited the chondrocyte differentiation and cartilage matrix synthesis by enhancing the Wnt5a expression. MiR-92a-3p regulates the cartilage development and homeostasis by directly targeting the Wnt5a, suggesting its important roles in OA pathology.

After IL-1β is added to rabbit bone marrow MSCs and chondrocytes cultured *in vitro*, chondrocyte viability decreases, cell apoptosis and mitochondrial membrane potential changes significantly. However, these changes disappeared after adding bone marrow MSC-derived exosomes. Compared with IL-1β group, the exosomes of bone marrow MSCs inhibit the phosphorylation of p38 and ERK, and promote the phosphorylation of Akt. These findings suggest that bone marrow MSC-derived exosomes inhibit the chondrocyte apoptosis through p38, ERK and Akt pathways (Qi et al., 2019).

MSCderived exosomes inhibit the pathogenesis of OA by inhibiting syndecan-1. Injection of exosomes containing miR-9-5p can alleviate inflammation and OA like injury, down-regulate the levels of inflammatory factors, alleviate the oxidative stress injury, and reduce the levels of OCN, MMP-13, comp and AKP. Syndecan-1 is the target of miR-9-5p. Up-regulation of syndecan-1 leads to aggravation of inflammation and OA like injury. The exosomal miR-9-5p derived from bone marrow MSC has anti-inflammatory and cartilage protective effects on OA by regulating the syndecan-1 (Jin et al., 2020a).

The expression of ELF3 increases and the expression of miR-136-5p decreases in traumatic OA cartilage. MiR-136-5p is confirmed to target the ELF3 and down-regulates its expression. After chondrocytes internalizes exosomes, the expression of ELF3 decreases. The exosomal miR-136-5p, derived from bone marrow MSCs, can promote the chondrocyte migration *in vitro* and inhibits the cartilage degeneration *in vivo*, thereby inhibiting the pathological changes of OA (Chen et al., 2020).

The stimulating effects of exosomes isolated from osteoblasts of coxarthrosis on bone marrow MSCs are mainly manifested in the catabolism and osteogenic differentiation. Interestingly, this has nothing to do with donor pathology, reflecting the influence of exosomes on tissue microenvironment and cell metabolism in coxarthrosis (Niedermair et al., 2020).

### Exosomes Derived From Primary Chondrocytes

The exosomes of primary chondrocytes may play a role in the treatment of OA. Zheng et al. (2019) isolated exosomes from primary chondrocytes cultured in normal and IL-1β induced inflammatory environment, and found that there were more mitochondrial proteins in exosomes of chondrocytes in normal group. Intra-articular injection of exosomes of chondrocytes in normal group successfully prevented the development of OA. Studies have shown that chondrocyte exosomes restored the mitochondrial dysfunction and promoted the macrophages to differentiate into M2 phenotypes.

Drug delivery is the key to the successful clinical application of nucleic acid drugs, and exosomal miRNAs in the treatment of OA has brought a new perspective to the treatment of OA. MiR-140 is not only significant in promoting the cartilage formation and inhibiting the degeneration, but also plays an important role in cartilage development (Duan et al., 2020). Liang et al. (2020) fused chondrocyte-affinity peptide (CAP) with lysosome associated membrane glycoprotein 2b on the surface of the exosomes to obtain cap exosomes. This exosomes could effectively encapsulate miR-140, specifically enter chondrocytes, and transport miR-140 to chondrocytes. Studies have shown that cap exosomes could also transfer miR-140 to the deep cartilage region through the dense mesochondral membrane, inhibited the cartilage degradation protease, and suppress the progress of OA.

MiR-95-5p regulates the chondrogenesis and cartilage degradation through the histone deacetylase (HDAC) 2/8. MiR-95-5p overexpression of primary chondrocyte derived exosomes may be effective in the treatment of OA. HDAC 2/8 is up-regulated in OA tissues and exosomes secreted by chondrocytes, and mediates the expression of specific genes in chondrocytes. MiR-95-5p directly acts on the 3′UTR of HDAC 2/8 to promote the cartilage formation and prevent OA by directly targeting the HDAC 2/8 (Mao et al., 2018a).

Transcription factor 4 (ATF4) plays an important role in chondrocyte proliferation and bone formation. It has been proved that the serum derived exosomes of OA mice has therapeutic effects on OA model mice (Chen D. et al., 2019). Studies have shown that intra-articular injection of ATF4-OA-Exosomes can reduce the degeneration, damage and inflammatory reaction of articular cartilage in OA model mice, and partially restore the autophagy function of knee cartilage. Furthermore, ATF4-OA-Exosomes promote the autophagy and inhibit the apoptosis in TNF-α or tunicamycin treated chondrocytes (Wang Y. et al., 2021).

### Exosomes Derived From Human Embryonic Stem Cells

The exosomes derived from human ESC-induced MSCs (ESC-MSCs) are involved in the therapeutic mechanisms of alleviating OA. In destabilization of the medial meniscus (DMM) model mice, intra-articular injection of ESC-MSCs reduces the cartilage destruction and matrix degradation in DMM model, which is mediated by ESC-MSCs derived exosomes. In the presence of IL-1β, these exosomes maintain the chondrocyte phenotype by increasing the type II collagen synthesis and decreasing the ADAMTS5 expression. Immunocytochemistry shows that the exosomes are colocalized with type II collagen positive chondrocytes. This provides a new target for the development of OA drugs and drug delivery systems (Wang et al., 2017).

### Exosomes Derived From Chondrogenic Progenitor Cells

Chondrogenic progenitor cells have high self-renewal ability and chondrogenic potential. Wang et al. (2020) found that intra-articular injection of exosomes secreted by chondrogenic progenitor cells in MRL/MpJ superhealer mice promoted the repair of articular cartilage injury in mice. By comparing the miRNA expression profiles of control CBA (CBA EVs) and MRL/MPJ mouse chondroblasts, the differentially expressed exosomal miRNAs were involved in a variety of biological processes. Among them, 80 miRNAs were significantly up-regulated and 100 were down-regulated, and 20 disordered miRNAs linked OA repair through the AMPK signaling, autophagy regulation and insulin signaling. The mechanisms of exosomes involved in OA may be more related to miRNAs (Toh et al., 2017).

### Exosomes Derived From Vascular Endothelial Cells

Exosomes from vascular endothelial cells have been proved to be involved in the pathogenesis of many diseases, and their roles in the OA pathogenesis have also been confirmed. Exosomes derived from vascular endothelial cells promote the pathological development of OA by inducing the chondrocyte apoptosis. These exosomes can inhibit the autophagy and p21 expression, reduce the ability of chondrocytes to resist the oxidative stress, increase the content of ROS and induce the apoptosis. Exosomes derived from vascular endothelial cells promote the progress of OA and provide new ideas for the diagnosis and treatment of OA (Yang et al., 2021).

### Exosomes Derived From Adipose-Derived Stem Cells

ADSCs are candidate cells for anti-inflammatory and cytoprotective effects on cartilage. Exosomes mediate the paracrine effect of ADSCs and down-regulate the aging characteristics of OA osteoblasts (Tofiño-Vian et al., 2017). ADSCs promote the chondrogenesis and inhibit the inflammation. Patients with OA are usually associated with obesity and chronic inflammation. Exosomes isolated from ADSCs down-regulate the expression of IL-6, NF-κB and TNF-α, and up-regulate the expression of IL-10. Exosome therapy can protect the articular chondrocytes from H2O2 induced apoptosis. In addition, exosome therapy promotes the chondrogenesis of periosteal cells and increases the level of chondrogenic markers, including type II collagen and β-catenin. Wnt signaling pathway may be its downstream signaling pathway. The periosteal cells with exosomes show high levels of miR-145 and miR-221, and the miR-145 and miR-221 are related to the enhancement of periosteal cells and chondrogenic potential, respectively (Zhao et al., 2020).

For exosomes derived from MSCs of infrapatellar fat pad (IPFP), miR-100-5p-abundant exosomes protect the articular cartilage and improve the gait abnormality by inhibiting the mTOR signal of OA. IPFP MSCs can produce a large number of exosomes, which show typical morphological characteristics of exosomes. IPFP MSC-derived exosomes can reduce the severity of OA, inhibit the apoptosis, promote the matrix synthesis and reduce the expression of catabolic factors. These exosomes can significantly enhance the autophagy level of chondrocytes through the mTOR inhibition. The detection of luciferase reporter gene shows that mir-100-5p combines with the mTOR’s 3′UTR, which reverses the mTOR signal pathway. It is important that intra-articular injection of antagormir-100-5p significantly reduces the protective effects of MSC-derived exosomes on articular cartilage. These IPFP MSCs are expected to be a potential therapy for OA (Clockaerts et al., 2010; Wu et al., 2019).

### Exosomes Derived From Human Dental Pulp Stem Cells

Exosomes of human DPSCs can inhibit the chondrocyte apoptosis in OA model rats. After transfection of DPSCs with miR-140-5p mimics, the exosomal miR-140-5p increased significantly. In IL-1β treated human chondrocytes, DPSC-derived exosomes promote the expression of chondrocyte related mRNAs, including aggrecan, Col2α1 and Sox9. Exosomes containing miR-140-5p significantly enhance this phenomenon. MiR-140-5p rich exosomes derived from DPSCs may play an anti-apoptotic role by regulating the expression of apoptosis related proteins. The exosomes of DPSCs may be a potential strategy for the treatment of OA (Lin et al., 2021).

Exosomes derived from stem cells of human exfoliated deciduous teeth (SHED) have a certain therapeutic effect on temporomandibular arthritis. MiR-100-5p is enriched in these exsomes. SHED exosomes inhibit the expression of IL-6, IL-8, MMP1, MMP3, MMP9, MMP13, disintegrin and metalloproteinase with thrombospondin motifs 5 (ADAMTS5). The chondrocytes treated with miR-100 mimics show low expression of the MMP1, MMP9, MMP13, ADAMTS5 and mTOR. On the contrary, miR-100 inhibition up-regulates these targets. Furthermore, miR-100-5p directly targets the 3′UTR of mTOR, and inhibits the expression of mTOR (Luo et al., 2019).

### Exosomes Derived From Monocyte Derived Cells

LncRNA MM2P and exosomes mediate the Sox9 transfer from monocyte derived cells to primary chondrocytes. Treatment of RAW264.7 mouse macrophages and mouse bone marrow-derived macrophages with IL-4 or IL-13 up-regulate the expression of MM2P. MM2P blocks the SHP2 mediated dephosphorylation of STAT3 at Try705 and interacts with RNA binding protein FUS. In turn, p-STAT3 increases the Sox9 gene expression. These cells release the Sox9 mRNA and exosomes containing proteins. The supernatant of these cells can promote the differentiation of primary chondrocytes, that is, up-regulates the expression of the Col1a2 and Acan genes, and promotes the secretion of extracellular matrix components. These effects are mediated by Sox9 mRNA and protein delivered to chondrocytes by exosomes. MM2P and its exosomes may be new therapeutic and diagnostic targets for OA (Bai et al., 2020).

### Exosomes Derived From Amniotic Fluid Stem Cells

The exosomes secreted by AFSCs have a certain effect on the treatment of OA. AFSCs can secrete exosomes with growth factors and immune regulatory molecules, which can prevent tissue degradation and induce the cartilage repair. Compared with the control group, the exosomes treated model animals show stronger pain tolerance and improved the histological score. Exosomes containing TGF-β can induce the cartilage recovery, which has better surface regularity and hyaline cartilage characteristics, and is positively correlated with the content of TGF-β. It is easier to detect macrophage markers in exosomes treated joints, which suggests that AFSC exosomes can regulate the macrophage polarization (Beretti et al., 2018; Zavatti et al., 2020).

### Exosomes Derived From Polydactyly Bone Mesenchymal Stem Cells

Zhou X. et al. (2020) obtained a special kind of MSCs from the bone marrow of patients with polydactyly, and found that polydactyly bone marrow-derived MSCs (pBMSCs) played certain roles in the pathological mechanisms of OA. It was important that pBMSCs have stronger ability to differentiate into chondrocytes than BMSCs. Exosomes secreted by pBMSCs stimulated the migration and proliferation of chondrocytes. The expression of BMP4 in pBMSCs was significantly higher than that in BMSCs, and the pBMSCs regulate chondrocyte formation through the BMP4 signal.

### Exosomes Derived From Mesenchymal Stem Cells of Temporomandibular Joint Osteoarthritis

In the immunocompetent model rats of TMJ-OA, MSC-derived exosomes play a key role in inflammatory response, injury behavior, condylar cartilage and subchondral bone healing. Exosomes mediated TMJ repair of OA is characterized by early inhibition of pain and degeneration, followed by reduced inflammation and sustained proliferation. MSC-derived exosomes gradually improve the matrix expression and subchondral bone structure, and promote the overall joint repair and regeneration. MSC-derived exosomes enhance the synthesis of s-GAG synthesis blocked by IL-1β, and inhibit the production of nitric oxide and MMP13 induced by IL-1β. Interestingly, adenosine receptor activation, Akt, ERK and AMPK phosphorylation inhibitors can partially eliminate these effects. Obviously, MSC-derived exosomes promote the repair and regeneration of TMJ in OA through a well coordinated mechanism (Cui et al., 2017; Zhang et al., 2019).

### Exosomes Derived From Mesenchymal Stem Cells of Lumbar Facet Joint Osteoarthritis

LFJ OA is one of the common causes of low back pain. In the try of mouse bone marrow MSC-derived exosome treatment, exosomes block the abnormal CGRP positive nerves and abnormal H-type angiogenesis in the LFJ subchondral bone, alleviating the low back pain. Bone marrow MSC-derived exosomes reduce the cartilage degeneration, inhibit the expression of tartrate resistant acid phosphatase, reduce the activation of RANKL-RANK-TRAF6 signal, and promote the subchondral bone reconstruction. Bone marrow MSC-derived exosomes have significant protective effects on patients with LFJ-OA, which may be a potential choice for the treatment of LFJ-OA (Li et al., 2020).

TGF-β1, transforming growth factor β1, regulates the proliferation of chondrocytes through MSC-derived exosomes. In the OA model, TGF-β1 stimulation enhances the expression of miR-135b in the MSC-derived exosomes and increases the survival rate of C5.18 cells. Interestingly, there is a negative regulatory relationship between miR-135b and Sp1. The combination of TGF-β1 and miR-135b inhibitor lead to the decrease of C5.18 cell activity. Obviously, TGF-β1 inhibits the SP1 through miR-135b derived from MSC-derived exosomes, and promotes the chondrocyte proliferation, and then promotes the cartilage repair (Wang et al., 2018).

### Effects of Exosomes on Macrophages in Osteoarthritis

Macrophages are derived from mononuclear cells in the blood after penetrating blood vessels. After entering connective tissue, the volume of monocytes increases, the endoplasmic reticulum and mitochondria proliferates, the lysosomes increases and phagocytic function enhances (Meng et al., 2019; Barrett, 2020). Macrophages have a series of continuous functional states. M1 and M2 macrophages are the two extremes of this continuous state. M1 macrophages participate in the positive immune response and play a role in immune surveillance by secreting pro-inflammatory cytokines and chemokines, and presenting antigens. M2 macrophages only have weak antigen presenting ability, and down-regulate the immune response by secreting inhibitory cytokines such as IL-10 or TGF-b, which play an important role in immune regulation. Macrophages play important regulatory roles in OA tissue repair, inflammatory response and chondrocyte proliferation (Wang S. et al., 2019; Russell et al., 2019).

Exosomes of OA chondrocytes promote the production of IL-1β in macrophages. These exosomes inhibit the LPS induced autophagy by inhibiting the expression of ATG4B through miR-449a-5p. The decrease of autophagy leads to the production of mitochondria, which further enhances the activation of inflammatory bodies and the subsequent production of IL-1β. This provides a new perspective for understanding the activation of synovial macrophages and OA pathogenesis in patients with OA (Ni et al., 2019).

MiR-135b is highly expressed in MSC-derived exosomes stimulated by TGF-β1. MiR-135b mimics induce the M2 polarization of synovial macrophases. The effects of miR-135b and TGF-β1-stimulated exosomes on the polarization of M2 synovial macrophases will be reversed by the increase of MAPK6. In conclusion, MSC-derived exosomal miR-135b promotes the polarization of M2 synovial macrophages by targeting the MAPK6, thus alleviating the cartilage damage and providing a new target for the treatment of OA (Wang and Xu, 2021).

OA is a chronic degenerative disease, which leads to limited activity and even disability. Exosomes derived from bone marrow MSCs can delay the progression of OA (Zhao et al., 2018). Exosomes reduce the cartilage damage and synovial macrophage infiltration, inhibit the M1 macrophage production and promote the M2 macrophage production. Exosomes reduce the expression of pro-inflammatory cytokines IL-1β, IL-6 and TNF-α in synovial fluid, and increase the release of anti-inflammatory IL-10. It is important that macrophages treated by exosomes maintain chondrogenic properties of chondrocytes. Obviously, bone marrow MSCs-derived exosomes alleviate the OA by promoting the phenotype transformation of synovial macrophages from M1 to M2 (Zhang et al., 2020).

The synovial exosomes stimulate the release of many inflammatory cytokines, chemokines and metalloproteinases by macrophages in OA, but do not affect the expression of CD80 and CD86 costimulator molecules. The purified exosomes has obvious functional activity in stimulating the release of pro-inflammatory factors by M1 macrophages (Domenis et al., 2017) (Table 1).

**TABLE 1:** Exosomes reported in the pathogenesis of OA.

Classification of exosomes	Exosomal inclusions	Origin of exosomes	Regulatory roles	Targets	References
Monocyte-derived exosomes	LncRNA MM2P	RAW264.7 mouse macrophages and mouse bone marrow-derived macrophages	Promotes the chondrocyte differentiation and functions	Sox9	Bai et al. (2020)
MSC-derived exosomes	MiR-136-5p	OA chondrocytes and mouse model of post-traumatic OA	Inhibits the chondrocyte degeneration in traumatic osteoarthritis	ELF3	Chen et al. (2020)
MSC-derived exosomes	MiR-127-3p	Bone marrow MSCs and primary chondrocytes of model rats	Inhibits the CDH11 in chondrocytes and relieving the chondrocyte damage in OA.	CDH11-mediated wnt/β-catenin pathway	Dong et al. (2021)
MSC-derived exosomes	MiR-9-5p	Rat model induced by anterior cruciate ligament/medial collateral ligament transection	Has anti-inflammatory and cartilage protective effects on OA	Syndecan-1	Jin et al. (2020a)
MSC-derived exosomes	MiR-26a-5p	MSC and synovial fibroblasts of OA model rats	Retards the damage of synovial fibroblasts *in vitro* and alleviates the OA damage	PTGS2	Jin et al. (2020b)
CAP exosomes	MiR-140	Chondrocytes of OA patients	Inhibits the cartilage-degrading proteases, and alleviates the OA progression in mode rats	Cartilage-degrading proteases	Liang et al. (2020)
Exosomes derived from mir-140-5p-overexpressing human DPSCs	MiR-140-5p	IL-1β treated human chondrocytes and OA model rats	Inhibits the chondrocyte apoptosis and improves the knee joint conditions in rat model	Chondrocyte apoptosis genes	Lin et al. (2021)
MSC-derived exosomes	LncRNA KLF3-AS1, miR-206	IL-1β-induced OA chondrocytes and collagenase-induced mouse OA model	Promotes the proliferation and inhibits apoptosis of chondrocytes	KLF3-AS1/miR-206/GIT1 axis	Liu et al. (2018a)
MSC-derived exosomes	LncRNA KLF3-AS1	IL-1β-induced OA chondrocytes and collagenase-induced mouse OA model	Exosomal KLF3-AS1 promotes the cartilage repair and chondrocyte proliferation	—	Liu et al. (2018b)
SHED-derived exosomes	MiR-100-5p	Temporomandibular joint chondrocytes	SHED-exosomes suppresses the inflammation in chondrocytes	mTOR	Luo et al. (2019)
Primary chondrocyte-derived exosomes	MiR-95-5p	OA primary chondrocyte	Promotes the cartilage formation and prevents the OA	HDAC2/8	Mao et al. (2018a)
MSC-derived exosomes	MiR-92a-3p	Human MSC and OA primary human chondrocytes	Enhances the chondrogenesis and suppresses the cartilage degradation via targeting WNT5A	WNT5A	Mao et al. (2018b)
Exosomes derived from OA patient serum and LPS-treated C28/I2 cells	LncRNA PVT1	OA patient serum and LPS-induced C28/I2 cells	Alleviates the lipopolysaccharide-induced OA progression	HMGB1/TLR4/NF-κB pathway via mir-93-5p	Meng et al. (2020)
Exosomes derived from osteoarthritic chondrocyte	MiR-449a-5p	Chondrocytes and macrophages of OA	Enhances the mature IL-1β production of macrophages and aggravates the synovitis in OA	ATG4B	Ni et al. (2019)
Exosomes derived from curcumin-treated MSCs	MiR-124 and miR-143	OA mouse models	Curcumin reinforces the MSC-derived exosomes in attenuating osteoarthritis	NF-kB and ROCK1/TLR9	Qiu et al. (2020)
Synovial MSC-derived exosomes	MiR-129-5p	OA patients and IL-1β-induced chondrocytes	Relieves the IL-1β induced OA	HMGB1	Qiu et al. (2021)
Synovial MSC-derived exosomes	MiR-140-5p	Human synovial MSCs	Enhances the cartilage tissue regeneration and prevents the OA in a rat model	RalA	Tao et al. (2017)
Exosomes derived from synovial fibroblasts	LncRNA H19	OA chondrocytes	Promotes the chondrocyte proliferation and migration and inhibits the degradation of OA matrix	MiR-106b-5p/TIMP2	Tan et al. (2020)
MSC-derived exosomes	MiR-135b	Chondrocyte of OA model rats	TGF-β1 promotes the chondrocyte proliferation by regulating the Sp1 through MSC-exosomes derived miR-135b	Sp1	Wang et al. (2018)
MSC-derived exosomes	MiR-135b	Cartilage tissues and synovial macrophages of model rats	Attenuates the cartilage injury via promoting the M2 synovial macrophage polarization	MAPK6	Wang and Xu. (2021)
Synovial MSC-derived exosomes	MiR-155-5p	OA chondrocytes and mouse model of OA	Prevents the OA via enhancing the proliferation and migration, attenuating the apoptosis, and modulating the ECM secretion	Runx2	Wang Z. et al. (2021
IPFP MSC-derived exosomes	MiR-100-5p	IPFP of OA patients	Protects the articular cartilage and ameliorates the gait abnormalities via inhibition of mTOR	3ʹUTR of mTOR	Wu et al. (2019)
ADSC-derived exosomes	MiR-145, miR-221	Chondrocytes of OA	Promotes the chondrogenesis and suppresses inflammation	Wnt/β-catenin	Zhao et al. (2020)
Exosomes derived from synovial fibroblasts	MiR-126-3p	Model rats of OA	Suppresses the chondrocyte inflammation and cartilage degradation	IL-1β, IL-6, and TNF-α	Zhou et al. (2021)

## The Roles of Exosomes in the Treatment of Osteoarthritis

Curcumin alleviates OA by enhancing MSC-derived exosomes. Curcumin significantly restores the expression of miR-143 and miR-124, and up-regulate the expression of NF-KB and ROCK1 in OA pathogenesis. The 3′UTR of NF-KB and ROCK1 contains the binding sites of miR-143 and miR-124, respectively. Importantly, curcumin reduces the DNA methylation of miR-143 and miR-124 promoters, suggesting that curcumin affects the methylation of these two miRNAs (Qiu et al., 2020).

Injection of bone marrow MSC-derived exosomes can alleviate the cartilage injury and pain in patients with OA. For example, exosomes treatment significantly attenuates the inhibitory effects of IL-1β on the chondrocyte proliferation and migration. Exosomes treatment significantly attenuates the IL-1β-induced down-regulation of COL2A1 and ACAN and up-regulation of MMP13 and ADAMT5. Exosomes treatment significantly reduces the up-regulation of CGRP and iNOS in dorsal root ganglion (DRG) of OA model rats. Compared with untreated OA model rats, the paw withdrawal latency (PWL) value of exogenous OA model rats was significantly increased (Liu et al., 2019).

Cosenza et al. (2017) compared the roles of bone marrow MSC-derived exosomes and microbubbles/microparticles (MPs) in OA pathogenesis. In OA chondrocytes, bone marrow MSC-derived exosomes and MPs both could inhibit the catabolism of MMP-13, ADAMTS5 and inflammatory marker iNOS, and re-induce the expression of aggrecan. Both exosomes and MPs could protect chondrocytes from apoptosis, inhibit the activation of macrophages, and protect model mice from joint injury. Exosomes and MPs replicate the main therapeutic effects of BM MSCs, suggesting that exosomes and MPs may contain the same substances that mediate intercellular communication.

In a bone defect treatment experiment, the osteochondral defect model was established in the trochlear groove of the distal femur of rats. One defect was treated with 100 μg human extracellular exosomes, and the exosomes were injected into the joint after operation. Once a week for 12 weeks. Compared with control, the appearance of the defect was enhanced and the histological score was improved. At 12 weeks, the defects treated with exosomes showed complete recovery of cartilage and subchondral bone, which confirmed the effectiveness of human embryonic MSC-derived exosomes in cartilage repair (Zhang et al., 2016).

MiR-155-5p overexpression of synovial MSCs *in vitro* prevents the OA by reducing the chondrocyte apoptosis and regulating the extracellular matrix (ECM) secretion of chondrocytes. Studies have shown that synovial MSC-derived exosomes promote the proliferation and migration of OA chondrocytes and inhibit their apoptosis, but has no effect on the secretion of ECM. MiR-155-5p overexpression in exosomes show common characteristics and further promote the ECM secretion by targeting the Runx2. Exosomal miR-155-5p effectively prevent the pathological development of OA model mice. Furthermore, overexpression of Runx2 partially reverses the effect of exosomal miR-155-5p on OA chondrocytes, proving that Runx2 is a direct target of miR-155-5p (Wang Z. et al., 2021).

Zhu et al. (2017) compared the efficacy of MSCs from synovial mesenchymal stem cells (SMMSC-Exos) and induced pluripotent stem cells (iMSC-Exos) in the treatment of OA. IMSC-Exos and SMMSC-Exos were injected into the joints of OA model mice induced by collagenase. The difference of therapeutic effects between them were evaluated by pathology, immunohistochemistry, cell count and scratch test. Both iMSC-Exos and SMMSC-Exos could reduce the symptoms of OA model in mice, but the therapeutic effects of iMSC-Exos were better than that of SMMSC-Exos, and iMSC-Exosomes promoted the migration and proliferation of chondrocytes more strongly. Since autologous iMSCs are inexhaustible in theory, iMSC-Exos may be a promising new method for the treatment of OA (Qiong et al., 2020).

## Conclusion and Perspectives

Exosomes are widely distributed in various body fluids, carrying and transmitting important signal molecules, forming a new intercellular information transmission system. Exosomes affect the physiological state of cells and are closely related to the occurrence and process of many diseases (Wang Y. et al., 2019). Almost all types of cells can secrete exosomes, and exosomes are also widely found in body fluids, including blood, tears, urine, saliva, milk, and ascites. At present, studies have found that exosomes contain the nucleic acids (miRNA, lncRNA, circRNA, mRNA, tRNA), protein, cholesterol, etc. The surface markers of exosomes are CD63, CD81, CD9, TSG101 and HSP70 (Li et al., 2018; Konečná et al., 2019). Therefore, exosomes can be associated with almost any disease and become an innovative hot spot in the research of disease biomarkers, disease mechanisms and drug development.

Exosomes transport proteins, mRNA, miRNA, lncRNA, circRNA, and even organelles into receptor cells, and participate in intercellular communication (Zhang et al., 2017). Exosomes play key roles in immune response, inflammation, angiogenesis, apoptosis, coagulation, waste disposal and other physiological processes. Exosomes from different cell sources contain different RNA and protein components, which can be used as early diagnostic biomarkers for a variety of diseases, and can also be used as carrier of drugs for disease treatment (Sung et al., 2018).

In addition, the characteristics of exosomes indicate its potential value in the treatment of OA. First, exosomes have a relatively long life span. Exosomes can be isolated from various body fluids and stored at −80°C for a long time. Secondly, exosomes carry bioactive substances, including mRNAs, miRNAs, lncRNAs and proteins, to protect them from enzymatic degradation, which indicates that exosomes have the potential to deliver nucleic acid and protein drugs to target cells. Third, exosomes can be further modified to carry specific drugs to meet the needs of specific treatment regimens. In this review, we provide evidence of exosomes in the direct and indirect regulation of OA pathogenesis, with particular emphasis on the roles of miRNAs and lncRNAs (Colao et al., 2018; Yamashita et al., 2018).

However, the potential feasibility and targets of exosomes as OA treatment vectors are not fully understood, and the basic and clinical research still has a long way (Lakshmi et al., 2021; Stefanius et al., 2021). In the future, the following problems must be solved. First, exosomes will be isolated and purified to eliminate the interference of vesicles and other cell metabolites. The targets and mechanisms of exosomes in different tissues need to be clarified, and the efficacy and safety of exosomes in different animal models need to be evaluated. Furthermore, we should pay attention to the importance of vesicles secreted by cells. Vesicles are not only the stumbling blocks of exosomes research, but also the next research hotspot, because vesicles may play a more special role than exosomes.

## Abbreviations

ADAMTS5, disintegrin and metalloproteinase with thrombospondin motifs 5; ADSCs, adipose-derived stem cells; AFSC, amniotic fluid stem cells; ATF4, transcription factor 4; CAP, chondrocyte-affinity peptide; Col2a1, collagen alpha 1; DMM, destabilization of the medial meniscus; E7 exo, E7 peptide; ESC, human embryonic stem cell; HDAC, histone deacetylase; HDAC3, histone deacetylase 3; IPFP, infrapatellar fat pad; KGN, kartogenin; lncRNAs, long noncoding RNAs; MSCs, mesenchymal stem cells; OA, osteoarthritis; pBMSCs, polydactyly bone marrow-derived MSCs; PHCs, primary human chondrocytes; PRP, platelet-rich plasma; SAGA, Spt-Ada-Gcn5 acetyltransferase; SHED, stem cells from human exfoliated deciduous teeth; SMMSC-Exos, synovial mesenchymal stem cells; TLR, toll-like receptor; TMJ-OA, temporomandibular joint osteoarthritis; YAP, yes-associated protein.
